# Unilateral Blurred Vision as the Sole Presenting Symptom of Chronic Lymphocytic Leukemia

**DOI:** 10.18502/jovr.v15i1.5958

**Published:** 2020-02-02

**Authors:** Rajesh K. Sharma, Kevin Mays

**Affiliations:** ^1^Department of Ophthalmology, University of Mississippi Medical Center, Jackson, MS, USA; ^2^Department of Neurology, University of Mississippi Medical Center, Jackson, MS, USA

**Keywords:** Chronic Lymphocytic Leukemia, Optic Neuropathy

## Abstract

**Purpose:**

To describe a case of infiltrative optic neuropathy caused by chronic lymphocytic leukemia.

**Case Report:**

A 41-year-old white male presented with painless, blurry vision in the left eye. Examination revealed unilateral optic nerve swelling confirmed by optical coherence tomography (OCT). Initial workup revealed mild leukocytosis, eventually diagnosed as chronic lymphocytic leukemia (CLL). No other cause of optic neuropathy was identified despite extensive investigation. The patient developed rapidly progressive retinal ganglion cell nerve fiber layer (NFL) atrophy and relative afferent pupillary defect (RAPD) of the left eye despite steroid treatment but stabilized after four cycles of CLL-targeted chemotherapy. Although infiltrative optic neuropathy is well-known in leukemia, presentation with only subtle vision loss is rare. Vision loss usually presents late in leukemic infiltrative optic neuropathy and therefore must be considered in patients with optic disc swelling and leukocytosis.

**Conclusion:**

When treating CLL, progressive visual decline with coexistent optic neuropathy may warrant chemotherapy.

##  INTRODUCTION

Optic neuropathy is the consequence of several pathologies including infection, systemic and localized inflammation, demyelinating disorders, and infiltrative conditions; however, it is most commonly the result of demyelination.^[1]^ Inflammatory neuritis usually presents as unilateral decreased vision with pain on ocular movement, but other neuropathies are frequently painless.^[2,3,4]^ Visual prognosis often depends on etiology and implementation of targeted treatment. It is therefore imperative to make all efforts to identify the cause; however, complex and mostly similar presentations of optic neuropathy makes the diagnosis difficult.^[3,5]^ Chronic lymphocytic leukemia (CLL) is a neoplastic expansion of B cells colonizing lymphoid tissues. In 2014, it was one of the most prevalent lymphoproliferative disorders in the United States, diagnosed at an observed incidence of 7.1 per 100,000 males according to the Surveillance, Epidemiology, and End Results (SEER) Program of the National Cancer Institute.^[1,2,6]^ Neuropathy caused by CLL via hematogenous infiltration of the optic nerve remains poorly described in literature with no clear guidelines for its management.^[1]^ Here, we present a case of a young male patient who was diagnosed with CLL following a subtle blurry scotoma; vision loss was stabilized following the treatment of his CLL.

##  CASE REPORT

A 41-year-old white male was referred to us for the evaluation of painless blurry vision in his left eye which had progressed gradually over the course of one month. The patient reported a constant inferonasal area of blurry vision with both near and distance vision. Otherwise, he had unremarkable ocular, medical, and surgical histories. He was an ex-cigarette smoker. He had a family history of non-melanoma skin cancer in his mother and multiple sclerosis in one distant relative. His review of systems, including additional neurological symptoms, was unremarkable.

**Figure 1:**
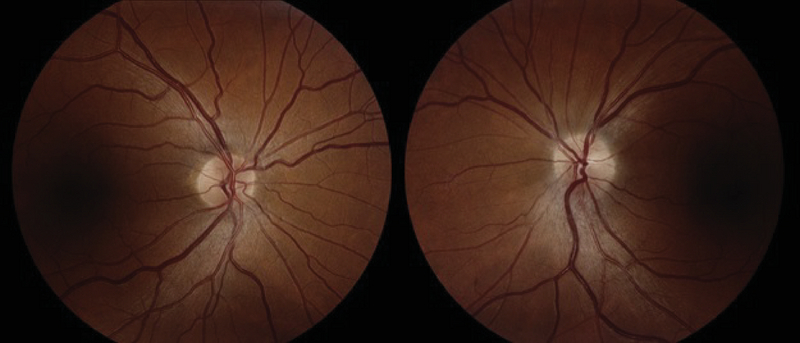
Color Fundus Photography taken at the Presentation. Disc margins are blurred along inferior border OS.

**Figure 2:**
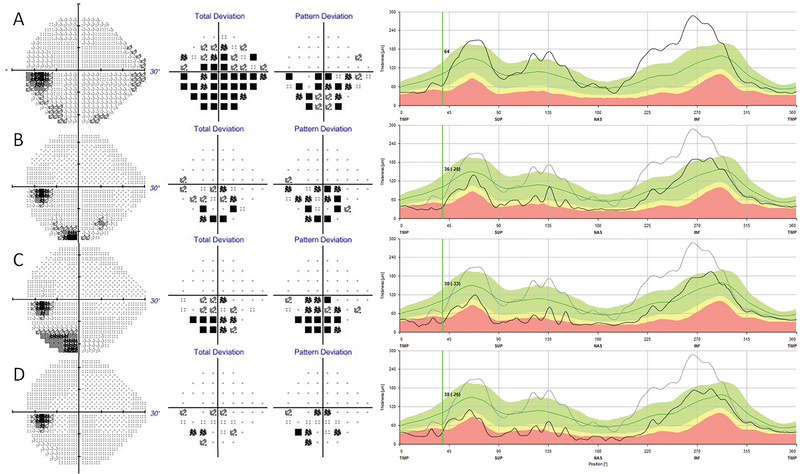
Static Perimetry Visual Field Testing with corresponding Optical Coherence Tomography Topographies. (A) Initial OS scotoma along inferior field on HVF 24-2 testing with corresponding baseline NFL thickness collected at the time of presentation. (B) Second visit HVF shows persistent scotoma and rapid NFL thinning compared to the baseline. (C) HVF during FR chemotherapy shows possible improvement of scotoma and arrest of NFL loss. (D) HVF several weeks post chemotherapy shows improvement in scotoma OS with stabilization of NFL loss compared to the baseline.

At the time of presentation, central visual acuity was 20/15 in his right eye (OD) and 20/25 in his left eye (OS). There was no overt dyschromatopsia on isochromatic color plates, although he had some difficulty reading the plates with his left eye. His pupils were equal and reactive without relative afferent pupillary defect (RAPD). Funduscopic examination of his left eye revealed inferior disc swelling which had not been present three years prior in his previous ophthalmologic exam. The initial laboratory results for unilateral optic nerve swelling included erythrocyte sedimentation rate (ESR), C-reactive protein (CRP), antinuclear antibody (ANA), angiotensin converting enzyme (ACE), rapid plasma reagin (RPR) levels, complete blood count (CBC), human immunodeficiency virus (HIV) titers, Bartonella antibody titers, Lyme disease panels, and herpetic viral panels. The results were normal except leukocytosis (19.7 TH/μL) with atypical lymphocytosis (74%) noted as abundant smudge cells. Serum polymerase chain reactions (PCR) for Epstein-Barr and cytomegalovirus were negative. Magnetic resonance imaging (MRI) of his brain and orbits, with and without contrast, was unremarkable. Static visual field testing (Humphrey Visual Fields: HVF) revealed an inferior scotoma in the left eye respecting the horizontal meridian. Initial color fundus photography [Figure 1] and Fourier-domain optical coherence tomography (OCT, Figure 2(a)) of the left eye showed inferior nerve fiber layer (NFL) swelling beyond his age-appropriate norm. The patient was admitted to the neurology ward for full workup and inpatient hematology consultation.

Collected cerebrospinal fluid (CSF) analyses, including opening pressure, infectious, autoimmune, and routine studies, were unremarkable. Repeat CBC with manual slide review confirmed the presence of leukocytosis with lymphocytic predominance, and fluorescent in situ hybridization (FISH) revealed 13q14 deletion. Flow cytometry showed monoclonal CD5+/CD10-B cell population consistent with CLL, classified as Rai stage 0/Binet stage A with favorable cytogenetic studies. Visual evoked potential was normal OD (106 milliseconds) but minimally prolonged OS (113 milliseconds). According to the National Comprehensive Cancer Network (NCCN) guidelines, the patient was not immediately treated for his CLL and was prescribed prednisone for suspected atypical infiltrative optic neuropathy.

One month after discharge, the patient reported stability of his left eye blurry scotoma without involvement of the right eye. He again denied focal neurologic symptoms. Physical examination showed stable central visual acuity, but identified new onset left RAPD; funduscopic examination re-demonstrated the left optic nerve inferior swelling unchanged from the previous exam. Static visual field testing showed slight worsening of his left eye inferior nasal scotoma and OCT revealed significant progression of NFL loss [Figure 2(b)]. Following discussions with our colleagues in the hematology department and extensive literature review, chemotherapy was initiated.

During the patient's first two cycles of FR chemotherapy, he noted no visual decline nor progression of his blurry vision scotoma. OCT during this time showed stabilization of the NFL without further loss [Figure 2(c)]. After four cycles of chemotherapy with continued stabilization of nerve fiber loss on OCT, and without progression of his scotoma size or density on HVF, we decided to monitor him according to the NCCN guidelines without further chemotherapy.

##  DISCUSSION

The differential diagnosis for optic neuropathy encompasses a wide range of systemic inflammatory, infectious, toxic, and inheritable diseases.^[1,2,5,7]^ The landmark Optic Neuritis Treatment Trial (ONTT) identified MRI as one of the most powerful tools in diagnosing optic neuritis; however, the identification of small inflammatory lesions can be limited by the resolution and sectioning of the MRI.^[5,8]^ Therefore, atypical optic neuritis must be considered even in the absence of MRI findings. In many cases, identifying the cause of optic neuropathy is a difficult endeavor.^[3,5]^ Extramedullary involvement of CLL is rare in reported literature, and due to the destructive nature of tissue sampling, optic nerve infiltration by atypical lymphocytes cannot be proven without postmortem biopsy.^[1,2,9]^ Even if the presence of CLL cells in the nervous system tissue is confirmed on biopsy, the presence of "bystander lymphocytes" can confound a presumed causative relationship.^[2]^ Infiltrative optic neuropathy by CLL has rarely been described in literature and these cases carry a higher risk of permanent unrecoverable loss of acuity, partially due to delay in diagnosis and treatment.^[1,9]^ We believe that the response to CLL treatment demonstrated by this patient, the absence of significant findings on imaging, and the exclusion of more common conditions as noted earlier corroborates that infiltrative CLL played a causative role in his optic neuropathy. Our observations also suggest that CLL treatment, including radiotherapy, should be considered in patients with suspected optic nerve infiltration to preserve vision, even if it is not indicated by genotyping.^[9]^

##  Financial Support and Sponsorship

Nil.

##  Conflicts of Interest

There are no conflicts of interest.
